# Enhanced and Sustainable Removal of Indoor Formaldehyde by Naturally Porous Bamboo Activated Carbon Supported with MnO_x_: Synergistic Effect of Adsorption and Oxidation

**DOI:** 10.3390/molecules29030663

**Published:** 2024-01-31

**Authors:** Zhenrui Li, Yujun Li, Shijie Li, Jianfeng Ma, Qianli Ma, Zhihui Wang, Jiajun Wang, Keying Long, Xing’e Liu

**Affiliations:** 1 International Centre for Bamboo and Rattan, Beijing 100102, China; lizr@caf.ac.cn (Z.L.); liyujun0225@163.com (Y.L.); lishijie@vision-smart.com (S.L.); majf@icbr.ac.cn (J.M.); maql@icbr.ac.cn (Q.M.); frankie2499@163.com (Z.W.); wjjchina0112@163.com (J.W.); 2 Key Laboratory of National Forestry and Grassland Administration/Beijing for Bamboo & Rattan Science and Technology, Beijing 100102, China; 3 Guangxi Zhuang Autonomous Region Forestry Research Institute, Nanning 530002, China; longky0305@163.com

**Keywords:** formaldehyde, bamboo activated carbon, manganese oxide, adsorption, catalytic oxidation

## Abstract

Novel bamboo activated carbon (BAC) catalysts decorated with manganese oxides (MnO_x_) were prepared with varying MnO_x_ contents through a facile one-step redox reaction. Due to the physical anchoring effect of the natural macropore structure for catalyst active components, homogeneous MnO_x_ nanoparticles (NPs), and high specific surface area over catalyst surface, the BAC@MnO_x_-N (N = 1, 2, 3, 4, 5) catalyst shows encouraging adsorption and catalytic oxidation for indoor formaldehyde (HCHO) removal at room temperature. Dynamic adsorption and catalytic activity experiments were conducted. The higher S_micro_ (733 m^2^/g) and V_micro_/V_t_ (82.6%) of the BAC@MnO_x_-4 catalyst could facilitate its excellent saturated and breakthrough adsorption capacity (5.24 ± 0.42 mg/g, 2.43 ± 0.22 mg/g). The best performer against 2 ppm HCHO is BAC@MnO_x_-4 catalyst, exhibiting a maximum HCHO removal efficiency of 97% for 17 h without any deactivation as RH = 0, which is higher than those of other MnO_x_-based catalysts. The average oxidation state and in situ DRIFTS analysis reveal that abundant oxygen vacancies on the BAC@MnO_x_-4 catalyst could be identified as surface-active sites of decomposing HCHO into the intermediate species (dioxymethylene and formate). This study provides a potential approach to deposit MnO_x_ nanoparticles onto the BAC surface, and this hybrid BAC@MnO_x_ material is promising for indoor HCHO removal at room temperature.

## 1. Introduction

Formaldehyde (HCHO) is a common toxic indoor air pollutant that originates from building materials, furniture, and home decorations [1,2]. HCHO pollution has received extensive attention and has been affirmed as carcinogenic to humans (Group 1) by the International Agency for Research on Cancer (IARC) because of its hazardous health effects, including irritation to human sense organs, headaches, or even immune dysfunction, and reproductive system abnormalities with long-term exposure to HCHO. Various methods have been developed to remove HCHO from indoor environments, including adsorption [3], catalytic oxidation [4], plasma destruction [5], photocatalysis [6], and botanical degradation [7]. However, achieving efficient indoor formaldehyde removal still faces significant challenges, primarily in two respects: (1) the indoor environment has a very low formaldehyde concentration, ranging from 0.1 to 1 mg/m^3^; (2) the airflow rate of formaldehyde in air filtration systems is as high as 1–3 m/s, resulting in a short residence time [8]. Constrained by ultra-low concentrations and high mass transfer rates, the removal efficiency of indoor formaldehyde still falls short of practical requirements. Moreover, according to the latest indoor air quality standards in China, the concentration of formaldehyde should not exceed 0.08 mg/m^3^ [9], which further brings challenges to the efficient removal of low-concentration HCHO. Among various methods, adsorption has commonly been considered as a high-efficiency method thanks to its economic applicability and facile operation [10].

Recently, biomass-based activated carbon (BBAC) has been used increasingly as a promising carbonaceous adsorbent and is expected to be an eco-friendly alternative to traditional activated carbon derived from non-renewable and expensive fossil fuels, such as coal-based and pitch-based activated carbon [11,12]. Compared to these environment-unfriendly resources, BBAC is derived from renewable agricultural and forest wastes, such as wood, bamboo, straw, stalk, and more, and has won the favor of researchers for its abundant feedstock, controllable cost, and satisfactory ability to remove gaseous pollutants (formaldehyde, acetone, and benzene) [13,14]. Nonetheless, the pristine BBAC adsorbents have a poor adsorption capacity for highly volatile and lightweight HCHO molecules due to their lack of surface affinity, which leads to secondary pollution during the regeneration process [15]. Therefore, it is necessary to develop improved methods that achieve multifunctional adsorption and degradation.

Composites fabricated with virgin adsorbents and catalytic active components can enhance HCHO degradation. Although supported noble metal catalysts have been proven to be effective, they are not widely applied due to their high price and limited supply [16]. Manganese oxides (MnO_x_), a relatively inexpensive transition metal oxide, have attracted attention thanks to their tunable crystal phases and low-temperature catalytic oxidation of HCHO [17,18]. As reported, manganese oxides have been proved to be more effective than TiO_2_, CeO_2_, or even some binary oxides for HCHO removal [19]. In particular, δ-MnO_2_, with a two-dimensional layered structure, has the highest catalytic activity among various crystal types of MnO_2_ [20]. However, when powdery MnO_2_ is used alone for HCHO removal in practical applications, its active components tend to aggregate, resulting in a significant reduction in active sites on the catalyst surface and a sharp decline in catalytic performance. Three-dimensional MnO_2_ frameworks were synthesized via the ice-templating method to continuously and efficiently transform HCHO into CO_2_ at room temperature [8]. Furthermore, supports with regular porous structures, as a kind of novel material, have recently received growing interest for achieving a good dispersion of catalyst components [21,22].

Bamboo activated carbon (BAC) is an eco-friendly material with a naturally rich porous structure that partially preserves the highly oriented and hierarchical gradient pore structure of natural bamboo [23,24]. This uniquely aligned three-dimensional pore structure enhances the internal medium diffusion, and the micro-channels inside (i.e., cell lumens and pits on the cell walls) benefit the uniform and sufficient deposition of a catalyst on the surface of the support material, which improves the overall performance of adsorption and catalytic oxidation over the composites. Therefore, bamboo catalysts are usually regarded as a suitable support material expected to decompose gas pollutants, such as HCHO [25], C_3_H_8_ [26], NO [27], and even for wastewater treatment [28].

The synergistic functions between BAC and MnO_x_ are defined as reactive adsorption and catalytic oxidation (RACO) [15]. MnO_x_/coconut shell AC composites have been assembled for the RACO of gaseous HCHO [29]. AC helps in the enrichment and adsorption of HCHO molecules, and MnO_x_ degrades HCHO molecules into innocuous formate (HCOO^−^) and carbonate (CO_3_^2−^) species, then further into H_2_O and CO_2_ via oxidation. Although numerous attempts have been made to study the catalytic oxidation mechanism for HCHO, the reactive adsorption mechanisms are not yet well understood.

Herein, a series of BAC/MnO_x_ composites with varying Mn contents were synthesized, and their performance of reactive adsorption and catalytic oxidation for indoor HCHO were evaluated at room temperature. The standard BAC samples with optimized pore structure activated by water vapor at 880 °C (supplied by the Jizhu Biological Technology Co., Ltd., Zhejiang, China) were used as carriers in this study. This type of BAC sample has a high specific surface area (~1061 m^2^/g) and microporosity (~985 m^2^/g) at a relatively low activation temperature and exhibits good economical applicability. Furthermore, in situ DRIFTS tests were employed to better understand the HCHO removal mechanism, especially the surface phenomena. This study provides an approach to develop a newly supported Mn-based catalyst for practical application.

## 2. Results and Discussion

### 2.1. Structural Characterization

**Figure 1:**
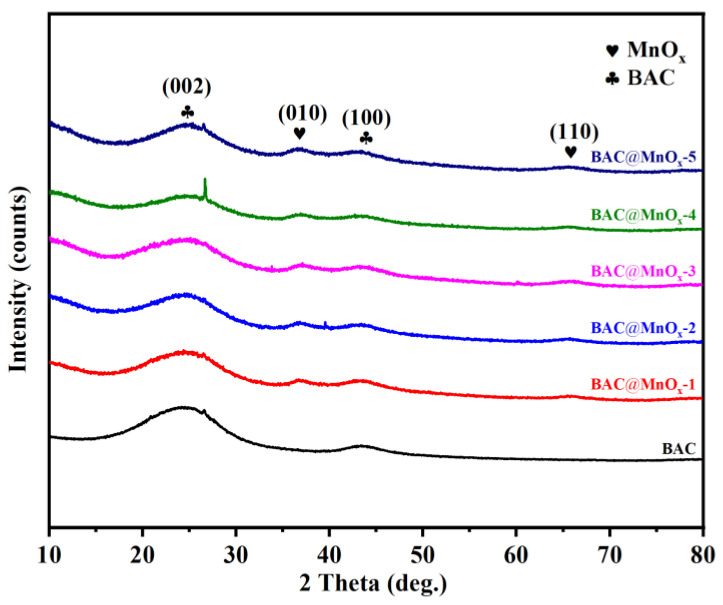
XRD patterns of BAC and BAC@MnO_x_-N (N = 1, 2, 3, 4, 5) series of catalysts.

Figure 1 displays the X-ray diffraction (XRD) patterns of BAC and BAC@MnO_x_-N (N = 1, 2, 3, 4, 5) series of catalysts. The broad diffraction peaks at 24.8° and 43.2° correspond to the (002) and (100) planes of highly graphitized carbon in BAC [30]. These two diffraction peaks became weaker in all catalysts with increasing Mn content, and the peak at the (002) plane slightly shifted to the right compared to BAC. This suggests a decrease in crystal plane spacing. This observation confirms the oxidative consumption of MnO_x_ to graphitized carbon, leading to the collapse of the layered structure of BAC. The weaker diffraction peaks around 36.6° and 65.4° were seemingly identified as (010) and (110) planes of the δ-MnO_2_ structure (JCPDS 80-1098) and Mn_2_O_3_ (JCPDS 00-031-0825), respectively [26,31]. In addition, a relatively sharp peak at approximately 26.6° was evident in all samples because the oxidative consumption exposed inorganic SiO_2_ to the sample surface [32].

**Figure 2:**
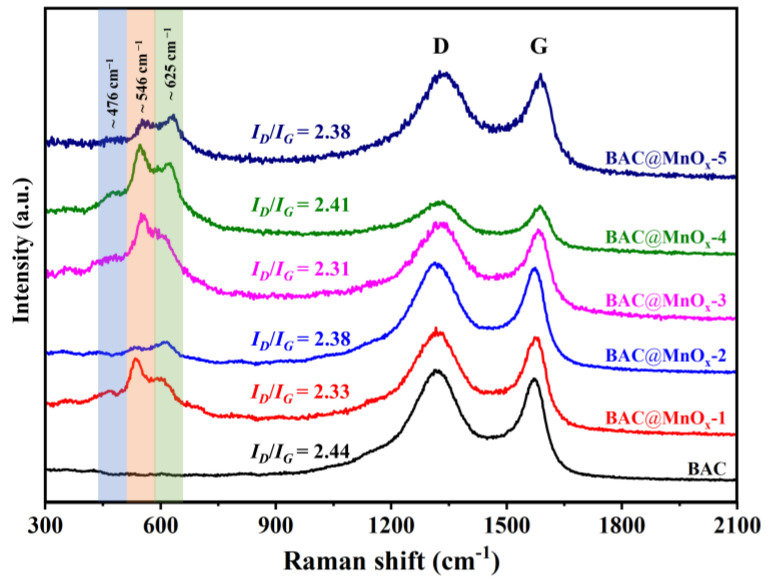
Raman spectra of BAC and series of catalysts.

Figure 2 shows the Raman spectra analysis of the BAC and all catalysts. The spectra of all catalysts exhibit three distinguishable peaks at around 476 cm^−1^, 546 cm^−1^, and 625 cm^−1^, which correspond to the Mn-O bond vibrations [8]. In particular, the peak at 476 cm^−1^ is attributed to the stretching vibration of Mn-O-Mn. The peak at 546 cm^−1^ is regarded as the Mn-O stretching vibration in the basal plane of the MnO_6_ sheet and is stronger in δ-MnO_2_ [33]. The peak at 625 cm^−1^ is assigned to the symmetric stretching vibration of Mn-O in MnO_6_. Among them, the BAC@MnO_x_-4 catalyst shows a significantly stronger peak at 546 cm^−1^, indicating a higher enrichment of MnO_x_ on the BAC surface. The fine analysis of Raman spectra at around 476 cm^−1^, 546 cm^−1^, and 625 cm^−1^ reconfirms the crystal structure of δ-MnO_2_. Additionally, the spectra exhibit two major bands at 1315 cm^−1^ (D band) and 1580 cm^−1^ (G band). The D band is associated with disordered carbon or graphitic structural defects, while the G band is connected with the graphitic and crystalline layers [34]. The intensity ratio of the D and G band (*I_D_*/*I_G_*) reflects the disorder degree of sp^2^ domains and the graphene defect [35]. For all catalysts, the *I_D_*/*I_G_* values were lower than that of BAC. This indicates a reduction in the disorder degree of the sp^2^ layered structure through the MnO_x_ decoration (Table S2).

**Figure 3:**
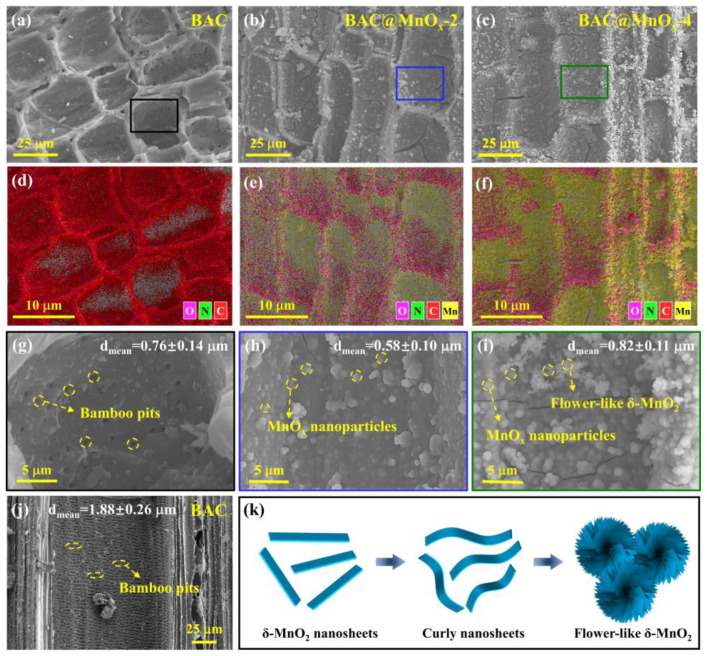
Typical SEM images of BAC (**a**,**g**), BAC@MnO_x_-2 catalyst (**b**), BAC@MnO_x_-4 catalyst (**c**), and its corresponding EDS elemental mappings (**d**–**f**) and area enlarged images (**g**–**i**), respectively. SEM image of bamboo vessels (**j**). A formation diagram of flower-like-MnO_2_ (**k**). The insets in (**g**,**j**) are the mean pore size of natural bamboo pits in the bamboo parenchyma cell and in vascular bundle system, respectively, after carbonization and activation processes, and the insets in (**h**,**i**) are the mean size of MnO_x_ NPs of BAC@MnO_x_-2 and BAC@MnO_x_-4, respectively. The black, blue, and green squares in (**a**–**c**) are enlarged and shown in (**g**,**h**).

The morphology of BAC and typical BAC@MnO_x_-2 and BAC@MnO_x_-4 catalysts was analyzed using the SEM technique, as shown in Figure 3. The virgin BAC sample has a relatively smooth surface with an abundant natural pore structure (Figure 3a). For the catalyst, it is clear that the flower-like MnO_x_ nanoparticles (NPs) are anchored on the BAC surface, and oxidative crack damage caused by permanganate ions reacting with the BAC sample was observed (Figure 3b,c). Specifically, spherical MnO_x_ NPs are self-assembled by irregular MnO_2_ nanosheets, which are likely to curl into pleated flower-like MnO_x_ (Figure 3k); then, they are bridged together to form a hierarchical porous architecture [36]. In addition, numerous pits spread over the inner surface of the bamboo parenchyma cell and the long protoxylem vessel channel in the vascular bundle system, which are enlarged during carbonization and activation processes, with a mean size of 0.76 ± 0.14 mm and 1.88 ± 0.26 mm, respectively. The hierarchical pore structure in the BAC sample with high flux is suitable for the efficient loading of MnO_x_ NPs and the formation of an interconnected catalytic network system (Figure 3g,j). Interestingly, the surface roughness of BAC increases with increasing Mn content, and more MnO_x_ NPs are observed over the catalyst sample. Compared to the BAC@MnO_x_-2 catalyst (0.58 ± 0.10 mm) (Figure 3h), the BAC@MnO_x_-4 catalyst with slight agglomeration has a higher average MnO_x_ NP size of 0.82 ± 0.11 mm (Figure 3i), which prevents MnO_x_ NPs inside the BAC surface from peeling off the smaller pits and guaranteed stable and abundant supply of catalytic active components. This is mainly due to the higher concentration of KMnO_4_, benefiting the formation of more crystal nuclei [37]. The MnO_x_ NPs with good dispersion are mainly formed on the surface of the BAC sample, which is supported by the EDS mapping results (Figure 3d–f).

**Figure 4:**
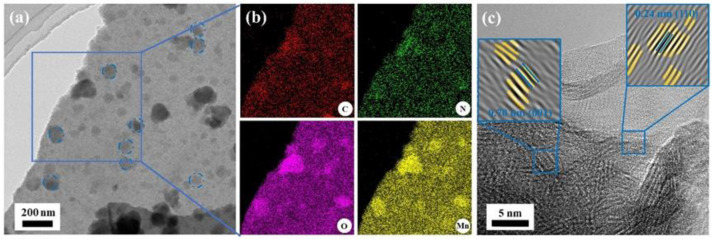
Representative HRTEM images (**a**,**c**) and corresponding elemental mapping images (**b**) of the blue square in (**a**) for the BAC@MnO_x_-4 catalyst. The insets in the blue square of (**c**) are the inverse FFT images of (001) and (110) lattice planes in the smaller blue square obtained from the original TEM image.

It is well known that sufficient loading and good dispersion of MnO_x_ are considered to be critical for effective HCHO removal. Among all the catalysts, BAC@MnO_x_-4 has a higher enrichment of Mn (10.44%) and appears to be a more homogeneous material, which is confirmed by HRTEM and corresponding elemental mapping as well (Figure 4). Uniform spherical NPs were observed, intermixed, and decorated with the amorphous BAC support (Figure 4a). Confirming the Raman spectra, the corresponding lattice space of 0.70 nm is assigned to the *d*-spacing of layered δ-MnO_2_, manifesting a lattice orientation of disordered arrangement, and the other lattice space of 0.24 nm is attributed to the (110) planes of δ-MnO_2_ (Figure 4c) [38]. Large nanosphere aggregates were not detected in the BAC@MnO_x_-4 catalyst, confirming the higher dispersion of δ-MnO_2_ on the catalyst surface. Therefore, it could be assumed that the BAC@MnO_x_-4 catalyst shows the greatest performance in HCHO oxidation removal.

**Figure 5:**
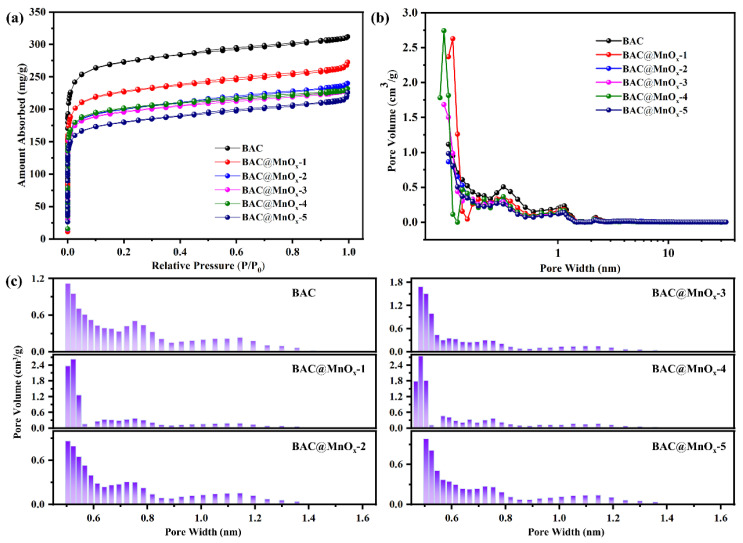
N_2_ adsorption/desorption isotherms (**a**) and pore size distributions (**b**) of the BAC and the series of catalysts. The amplification area of pore size distribution in the micropore range (**c**).

The N_2_ adsorption/desorption isotherms at 77 K, pore parameters of BAC, and all catalysts are presented in Figure 5 and Table 1. All samples exhibit a type I isotherm with non-significant type H4 hysteresis loops when P/P_0_ is over 0.5. When P/P_0_ is below 0.1, the N_2_ adsorption volume increases sharply, indicating that there are a large number of micropores and trace mesopores (Figure 5a). Moreover, the adsorption capacity of all catalysts for N_2_ gradually decreases with an increase in the amount of Mn loading resulting from the agglomeration of MnO_x_ NPs, particularly for the BAC@MnO_x_-5 catalyst and, further, reducing the number of effective adsorption sites. This can also be observed due to the decline in the *S*_BET_ and *V*_t_ of all catalysts, as compared to BAC. As shown in Table 1, compared to the virgin BAC (1061 m^2^/g, 0.48 cm^3^/g), the *S*_BET_ and *V*_t_ of BAC@MnO_x_-N (N = 1, 2, 3, 4, 5) catalyst decrease from 879 to 696 m^2^/g and from 0.42 to 0.35 cm^3^/g, respectively. Among all catalysts, despite the large amount of Mn loading (10.44%) and the maximum mean particle size (0.75 mm), the BAC@MnO_x_-4 catalyst exhibits a relatively higher *S*_BET_ (783 m^2^/g) and the lowest *D*_av_ (0.89 nm). This is because the uniform distribution of MnO_x_ NPs in the BAC@MnOx-4 catalyst is stacked in layers, creating new microporous structures on the surface instead of merely filling the micropores of the BAC substrate material.

In addition, the PSD in the micropore range (D_pore_ < 2 nm) of all samples reveals hierarchical microporous structures, and there is no significant mesoporous structure (2 nm < D_pore_ < 50 nm) (Figure 5b). The micropore system of virgin BAC was mainly concentrated at around 0.50 nm and 0.75 nm, respectively. By contrast, after MnO_x_ loading, some catalysts exhibited an increased proportion of ultra-micropores (D_pore_ < 0.7 nm) [39]. Among them, the BAC@MnO_x_-4 catalyst had the most developed micropores, containing three ultra-micropore systems, mainly around 0.47, 0.48, and 0.50 nm (Figure 5c). Generally, the larger *S*_micro_ and the ratio of *V*_micro_ to V_t_, the more active sites and larger interface for the adsorption and the following HCHO oxidation on BAC@MnO_x_ catalysts, thereby promoting the generation of a highly synergistic adsorption–oxidation performance [40].

### 2.2. XPS

**Figure 6:**
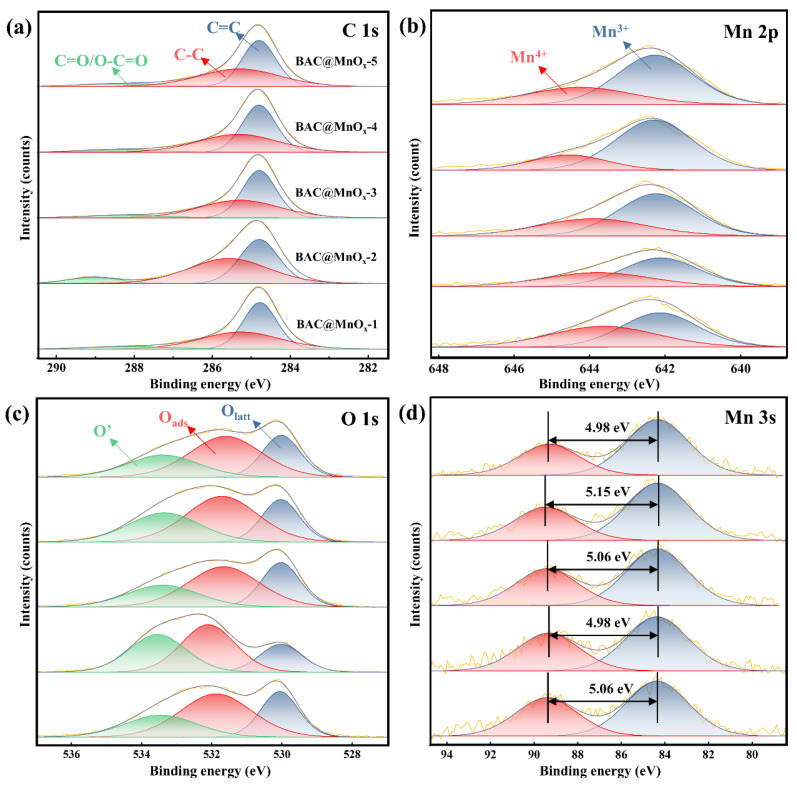
XPS spectra of C1 s (**a**), Mn 2p (**b**), O 1s (**c**), and Mn 3s (**d**) series of catalysts. The spectra in each (**b**–**d**) represent the BAC and the series of catalysts, respectively, similar to the (**a**). The number in (**d**) is the difference between two multiple splitting seams of Mn 3s.

XPS spectra were utilized to identify the surface chemical composition of all catalysts, as presented in Figure 6. The typical signals for C, O, and Mn are clearly presented in the full-range spectra (Figure S2), and the quantitative analysis was calculated and is listed in Tables S3 and S4. Generally, the C 1s spectra are deconvoluted into three peaks at 284.8, 285.3–285.6, and 288.1–289.0 eV (Table S3), which are assigned to the species of sp^2^ C=C, sp^3^ C-C, and C=O, respectively (Figure 6a) [41,42]. The Mn 2p_3/2_ spectra are deconvoluted into two peaks with binding energies of 643.6–644.6 and 642.1–642.3 eV, identifying Mn^4+^ and Mn^3+^ species, respectively (Figure 6b and Table S3) [43]. The coexistence of Mn^3+^ and Mn^4+^ species might enhance the oxidation of HCHO or other intermediates through electron transfer and conversion [44]. Compared to the other catalysts, the higher ratio of Mn^3+^/Mn^4+^ (3.27) of the BAC@MnO_x_-4 catalyst means more oxygen vacancies are required to maintain electrostatic balance (Table 2). The average oxidation state of Mn originating from the Mn 3s spectra also confirms this result (Figure 6d). The BAC@MnO_x_-4 catalyst possesses a different binding energy (ΔE) of 5.15, and the corresponding average oxidation state of Mn is 3.16 [45]. Furthermore, the O 1s spectra were fitted into three peaks at 530.0–531.1, 531.6–532.1, and 533.4–533.6 (Figure 6c and Table S3). The BAC@MnO_x_-4 catalyst has a higher O_ads_/O_latt_ ratio (2.04) than the other catalysts (Table 2), indicating a higher content of surface-adsorbed oxygen species (O_2_^−^, O^−^, etc.). This is considered one of the reasons for its high activity [45]. Therefore, it can be assumed that the BAC@MnO_x_-4 catalyst is endowed with the most enhanced activity for HCHO oxidation due to the abundant surface-adsorbed oxygen species.

### 2.3. Redox Properties

**Figure 7:**
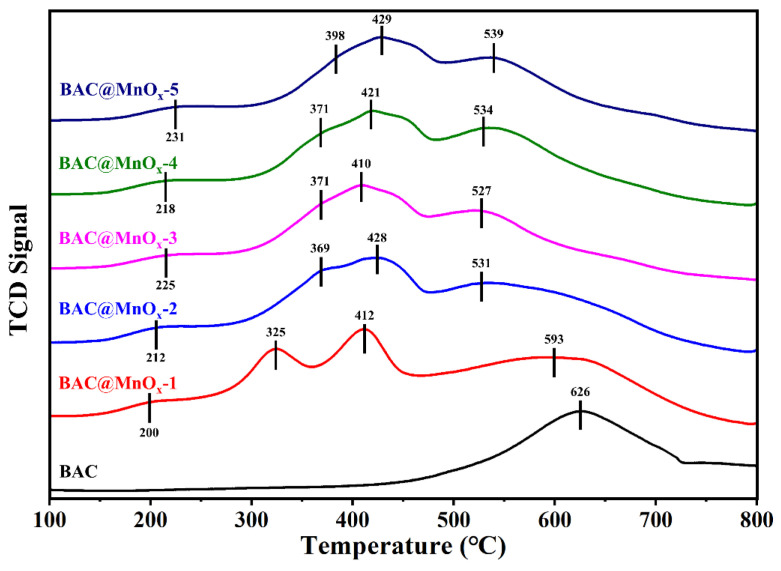
H_2_-TPR profiles of the BAC and the series of catalysts.

The redox properties of the BAC and all catalysts were evaluated using H_2_-TPR experiments, and the results are presented in Figure 7. For BAC, the H_2_-TPR profile shows a broad peak, characterized by an apparent maximum at around 626 °C, which was assigned to the gasification of the carbon support [46]. After loading MnO_x_, there were mainly three obvious reduction peaks at 325–398 °C, 412–429 °C, and 527–593 °C for all catalysts, which was attributed to the gradual reduction of MnO_2_ to Mn_2_O_3_/Mn_3_O_4_ and finally to MnO [47,48]. In addition, all catalysts show small peaks at around 200–231 °C, corresponding to the reduction of easily reducible surface-adsorbed oxygen species (e.g., O_2_^−^ and O^−^) [49]. Moreover, the BAC@MnO_x_-1 shows a lower initial reduction property as compared to other catalysts. However, other catalysts exhibit a lower reduction temperature at around 527–539 °C, significantly lower than that of the BAC@MnO_x_-1 (593 °C) with the increasing temperature, indicating higher redox properties and the formation of more oxygen vacancies according to the ratio of Mn^3+^/Mn^4+^ XPS results [50]. The strong interaction between MnO_x_ and the carbon support could be inferred from the higher redox properties. This strong interaction can enhance the mobility of active oxygen species over the surface of catalysts, promoting the decomposition of VOCs and the further oxidation of byproducts [51].

### 2.4. Evaluation of Performance in HCHO Removal

#### 2.4.1. Adsorption Capacity

**Figure 8:**
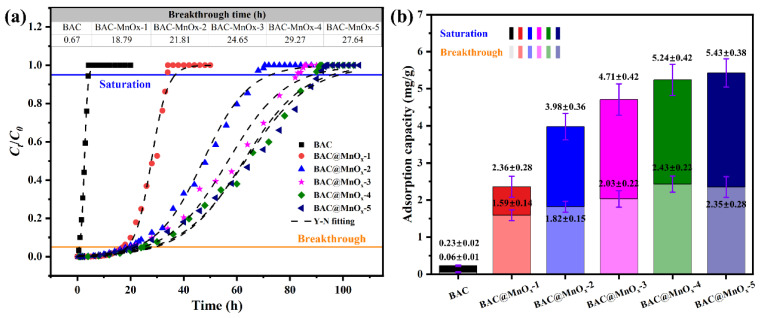
Breakthrough curves (**a**) and adsorption capacity (**b**) under ambient conditions (25 ± 3 °C and RH = 0%) of BAC and all catalysts. The inset table in (**a**) is the breakthrough time of BAC and all catalysts calculated by the Y-N model.

To evaluate the adsorption contribution of activated carbon in catalysts, their adsorption capacity and breakthrough curves were measured in ambient conditions (25 ± 3 °C and RH = 0%), as shown in Figure 8. The breakthrough curves of the BAC and all catalysts were well fitted by the Yoon–Nelson (Y-N) model (R^2^ = 0.990–0.995) (Table S4). Compared to BAC (0.67 h, 0.06 mg/g), the breakthrough time and adsorption capacity of catalysts increased significantly by at least 27% and 26.5%, respectively, even with a small amount of catalyst content (e.g., BAC@MnO_x_-1). In addition, the saturated adsorption capacity of all catalysts also increased with increasing Mn content, while the breakthrough time and adsorption capacity did not always increase. With increasing Mn content, the breakthrough time and adsorption capacity first increase and then decrease. The highest values were observed for BAC@MnO_x_-4 of 29.27 h and 2.43 mg/g, respectively. Therefore, it could be concluded that the pure commercial BAC exhibits an inferior adsorption performance while providing abundant macropores for MnO_x_ loading and micropores for HCHO adsorption.

#### 2.4.2. Catalytic Oxidation Performance

**Figure 9:**
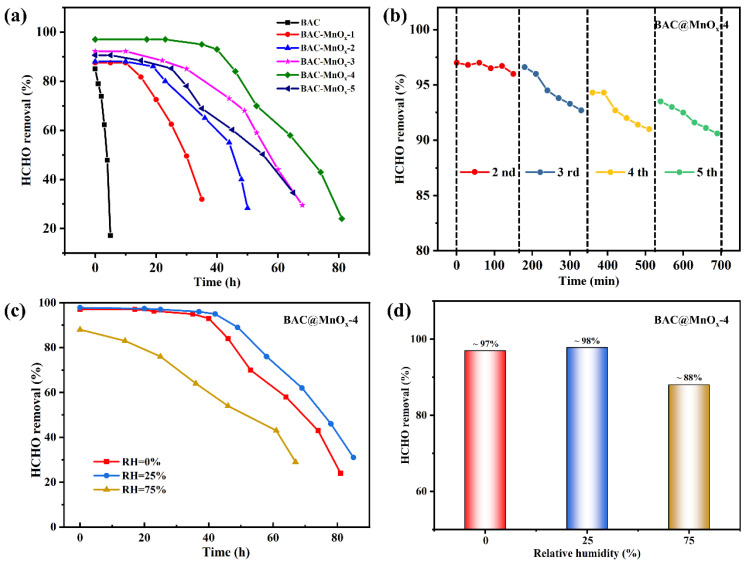
HCHO removal efficiency of the BAC and the series of catalysts over time (**a**). Stability tests (**b**) and humidity effect (RH = 0, 25, and 75%) on the BAC@MnO_x_-4 (**c**,**d**) in dynamic tests. Testing condition of (**a**,**b**): RH = 0%.

The catalytic activity, cycling stability, and water resistance for HCHO oxidation over BAC and all catalysts are presented in Figure 9. The BAC shows a transient property, with a maximum HCHO removal efficiency of approximately 85%, which sharply decreases to 17.2% within 5 h. This indicates limited adsorption capacity. In contrast, the HCHO oxidation performance of all catalysts improves considerably with a focus on catalytic efficiency and persistence. The catalytic activity is significantly influenced by the Mn content. Specifically, the highest HCHO removal efficiencies within the first 5 h were ~88%, ~88%, ~92%, ~97%, and ~91% for BAC@MnO_x_-1, BAC@MnO_x_-2, BAC@MnO_x_-3, BAC@MnO_x_-4, and BAC@MnO_x_-5, respectively (Figure 9a). The oxidative activity drops to different extents afterward, with the highest values for BAC@MnO_x_-4. HCHO removal efficiency is maintained at 97% after 17 h, which is superior to that reported in the literature (Table 3). This superior performance might be due to the better electron transfer capacity of the relatively homogeneous structure of this hybrid material and the sufficient decoration by MnO_2_ nanosheets/nanospheres. The cycling stability of the catalysts at room temperature is also excellent, with BAC@MnO_x_-4 maintaining a high HCHO removal efficiency (90.6%) after five cycles within almost 700 min (Figure 9b). Moreover, the presence of H_2_O greatly influences the catalytic ability to scavenge HCHO, particularly in highly humid conditions. The HCHO removal efficiency of BAC@MnO_x_-4 was found to be only slightly higher under RH = 25% (~98%) compared to that under RH = 0% (~97%). However, when exposed to a higher relative humidity of RH = 75%, the HCHO removal efficiency shows a sharp decline from 88% to 83% within 14 h. (Figure 9c,d). This suggests that water steam not only adsorbs onto the catalyst surface and replenishes the surface -OH groups consumed during HCHO oxidation under lower-humidity environments but also pre-empties the catalytic active sites, leading to a decrease and even deactivation of the catalytic activity in higher-humidity environments.

### 2.5. Mechanism of HCHO Oxidation

**Figure 10:**
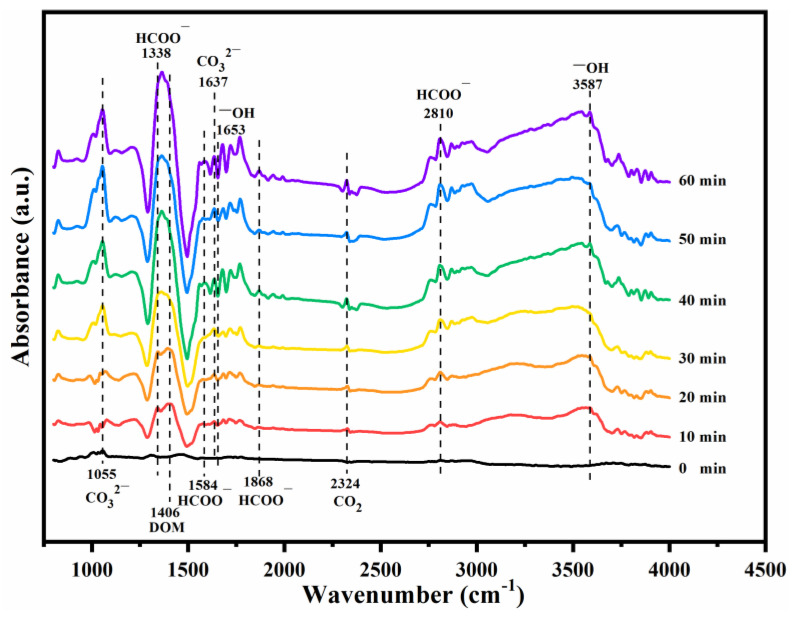
In situ DRIFTS spectra of the BAC@MnO_x_-4 catalyst exposed to 30 mL/min 100 ppm HCHO/O_2_ at room temperature.

In situ DRIFTS spectra were further collected to study the degradation pathway and the intermediate species produced by the reaction process over the typical BAC@MnO_x_-4 catalyst at room temperature. As shown in Figure 10, absorption bands at around 1720–1740 cm^−1^ are generally attributed to the aldehyde group of HCHO [56]. Thus, the physical adsorption for HCHO removal contributes significantly by gathering the HCHO molecules on the BAC surface. Following the introduction of HCHO gas, absorption bands at 1055, 1338, 1406, 1584–1653, 1868, 2324, 2810, and 3587 cm^−1^ were detected, attributed to the carbonate species (CO_3_^2−^) at 1055 and 1637 cm^−1^, formate species (HCOO^−^) at 1338, 1584, 1868, and 2810 cm^−1^, the dioxymethylene (DOM) species at 1415 cm^−1^, the surface hydroxyl groups (-OH) at 1653 cm^−1^ and 3587 cm^−1^, and CO_2_ bands at 2324 cm^−1^, respectively [57]. The generation of these intermediate species and CO_2_ confirms that the reaction process is both physical adsorption and the chemical decomposition of HCHO.

Specifically, within the first 30 min of reaction, the peak intensity of intermediate species continuously increases with decreasing surface -OH at 1653 cm^−1^. Meanwhile, the peak intensity of surface -OH compensated from the surface-adsorbed water at 3587 cm^−1^ gradually increases. This confirms the formation and decomposition of these intermediate species-consumed -OH groups that are further replenished by the surface-adsorbed water. As the reaction progresses, the peak intensity of the intermediate species basically remains stable, suggesting a dynamic balance between the generation and desorption of intermediates and no accumulation over the catalyst surface, which is responsible for remarkable activity and stability of the BAC@MnO_x_-4 catalyst in HCHO degradation [58].

**Figure 11:**
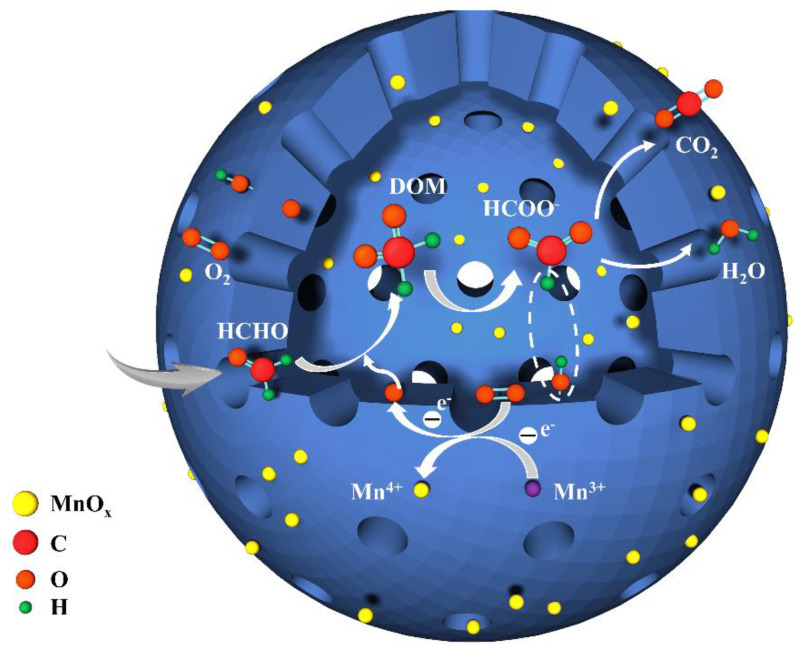
Mechanism of HCHO degradation on the multifunctional BAC@MnO_x_ catalyst.

Based on the above results, we propose a potential mechanism for the oxidative decomposition of HCHO on the BAC@MnO_x_ catalyst. The introduction of a naturally porous BAC adsorbent enhances the dispersion of MnO_2_ and the homogeneity of the composite materials, resulting in the creation of more efficient active sites, both inside and outside the catalyst surface. Additionally, the natural macropores of BAC serve as an anchor for the catalyst components, preventing their detachment from the internal surface of BAC and ensuring remarkable catalytic activity and cyclic stability during HCHO degradation. As depicted in Figure 11, HCHO molecules are diffused into the inner structure of BAC, initially through the natural macropore channels and, subsequently, are adsorbed onto the internal and external surfaces of BAC via hydrogen bonding and the active MnO_2_ component surface.

Simultaneously, gaseous oxygen molecules are captured by the oxygen vacancies on the catalyst surface and undergo dissociation to activate reactive oxygen species (O_2_^−^, O^−^). The adsorbed HCHO molecules on the catalyst surface are initially oxidized to an intermediate species (DOM) by the reactive oxygen species. DOM, highly unstable, further decomposes into H^+^ and HCOO^−^, which are then broken down into CO_2_ and H_2_O by active hydroxyl groups and reactive oxygen species. The consumed surface reactive oxygen species are replenished through an oxidation–reduction cycle involving Mn^3+^ and Mn^4+^ species and molecular oxygen. Similarly, the consumed active hydroxyl groups can be replenished by adsorbed water on the surface of activated carbon. Thus, the outstanding catalytic activity and stability observed in the decomposition of formaldehyde by the BAC@MnOx-4 catalyst can be attributed to the abundant and uniformly distributed catalytic active components, along with the excellent HCHO adsorption capacity and stable anchoring effect provided by the BAC pore structures.

(1) $$O_{2}\overset{MnO@BAC}{\to }O_{2^{-}}+O^{-}$$

(2) $$HCHO+O_{2^{-}}/O^{-}\to DOM$$

(3) $$DOM\to H^{+}+{HCOO}^{-}$$

(4) $$H^{+}+{OH}^{-}\to H_{2}O$$

(5) $${HCOO}^{-}+O_{2^{-}}/O^{-}/{OH}^{-}\to H_{2}O+{CO}_{2}$$

## 3. Materials and Methods

### 3.1. Materials

Commercial BAC sample was purchased from Jizhu Biological Technology Co., Ltd. (Zhejiang, China). Potassium permanganate (KMnO_4_) was bought from Sinopharm Chemical Reagents Co., Ltd. (Shanghai, China). The solid paraformaldehyde particles and the formaldehyde water solution (37 wt% in water) were bought from Aladdin Reagent Co., Ltd. (Shanghai, China). Deionized water was purified in the laboratory, and its conductivity was less than 0.5 us/cm. All chemicals were used as analytical reagents without further purification.

### 3.2. Catalyst Synthesis

BAC powder (40–60 mesh) was washed with deionized water repeatedly and boiled for 30 min to remove ash particles. The resulting BAC catalyst support has a final ash content of 1.49% after filtering and drying at 80 °C for 12 h. Catalyst preparation was carried out using an in situ one-step reduction method. To prepare the catalysts, different amounts of KMnO_4_ (0.571 g, 1.143 g, 1.714 g, 2.286 g, and 2.857 g) were dissolved in 100 mL of deionized water. Then, 2 g of BAC was added to each KMnO_4_ solution and stirred before it was filtered and dried at 80 °C for 12 h, respectively. Finally, the MnO_x_ active components were deposited into the BAC surface, according to the following redox reaction:

4MnO^4−^ + 3C + H_2_O → 4MnO_2_ + CO_3_^2−^ + 2HCO^3−^

The resulting catalysts were labeled as BAC@MnO_x_-N (N = 1, 2, 3, 4, 5) based on the amount of KMnO_4_ used, with actual Mn content of 7.72%, 8.98%, 10.24%, 10.44%, and 11.04%, respectively (Table S1). A control sample was prepared using BAC only without MnO_x_ support.

### 3.3. Catalyst Characterization

The crystal structure of as-prepared powdered catalysts (200 mesh) was analyzed via X-ray diffractometer (XRD, UItima IV, Rigaku Corporation, Akishima-shi, Tokyo, Japan) with a Bragge–Brentano geometry using Cu-Kα radiation (λ = 0.15418 nm) with the 3 kW of output rating and a step size of 5°/min in the 2θ range from 10° to 80°. The XRD results were analyzed using Jade 6.5 software. The structure defect of catalysts was characterized by a microscopic confocal Raman spectrometer (Lab RAM HR800, France) with an excitation wavelength of 532 nm. Raman signals were detected by a charge-coupled device (CCD) detector system. The Mn element content in the catalysts was tested by inductively coupled plasma atomic emission spectrometry (ICP-AES, Agilent 5110, Palo Alto, CA, USA). The morphology and element distribution of catalysts were examined using field-emission scanning electron microscopy (SEM-EDAX, HORIBA X-Max, Kyoto, Japan) at 200 kV, equipped with an energy-dispersive X-ray spectrometer. The microstructure of catalysts was characterized by field-emission gun source-based high-resolution transmission electron microscopy (HRTEM, JEM-F200, JEOL, Rigaku Corporation, Akishima-shi, Tokyo, Japan) with elemental mapping analysis and an acceleration voltage of 200 kV. The lattice spacing was observed on HRTEM bright-field images with a lattice resolution of 0.19 nm. The characteristics of the pore structures of the catalysts were measured through N_2_ sorption isotherms at 77 K using a nitrogen adsorption–desorption apparatus (Anton Paar, Autosorb IQ, FL, USA). The specific surface area (SSA) and pore structures were calculated based on the Brunauer–Emmett–Teller (BET) method. The pore size distribution was estimated using the density functional theory (DFT) method. The elemental compositions of the catalyst surface were analyzed using X-ray photoelectron spectrometer (XPS, Thermo Fisher Scientific, Waltham, MA, USA) with Al Kα X-ray source at 160 eV pass energy. The binding energy results of each catalyst were calibrated with C 1s = 284.8 eV as reference. H_2_ temperature-programmed reduction (H_2_-TPR) was performed to analyze the redox capacity on a Micromeritics AutoChem II 2920 apparatus with 100 mg of the catalyst with 10% H_2_/Ar mixed gas flow at a rate of 50 mL min^−1^. The reduction curve was collected at temperatures ranging from 30 to 800 °C at a rate of 10 °C/min. The species and content changes in the intermediate during catalytic oxidation reaction were collected by in situ diffuse reflectance infrared Fourier-transform spectrometry (In situ DRIFTS, Thermo IS50, Waltham, MA, USA). The spectra in the region from 4000 to 1000 cm^−1^ were recorded at 4 cm^−1^ resolution and 128 scans.

### 3.4. Catalytic Activity and Adsorption Performance for HCHO Removal

The dynamic adsorption and catalytic activity test for HCHO removal were evaluated in a fixed continuous thermostatic fixed-bed reactor at temperature of 25 °C. The HCHO adsorption system consisted mainly of three parts: a gas generator, an adsorption column part, and an analysis system (as shown in Figure S1). The dry air was divided into three flows by three mass flow meters. Two of the three gas flows were guided into two bubblers containing deionized water and solid paraformaldehyde particles, respectively, and the third gas flow was dry air. HCHO vapor was generated by passing dry air through solid paraformaldehyde particles in bubbler at 30 °C, maintained with the aid of the water bath, and HCHO vapor and dry air were then introduced to a gas mixing chamber to adjust the inlet concentration. The concentration of HCHO was controlled at around 1.4 mg/m^3^ (~2 ppm) with a flow rate of 200 mL/min in the gas current leading to the adsorption column part, which is a cylindrical glass tube with an internal diameter of 6 mm and 3 cm in length and contains 0.2 g of catalyst; the corresponding space velocity (GHSV) is ~60 L/g_cat_·h. The inlet and outlet concentrations of HCHO were determined by MBTH method implemented in an ultraviolet and visible spectrophotometer (UV-3600Plus, Shimadzu, Japan) [52]. The HCHO removal efficiency (*η*) was calculated according to Equation (6):

(6) $$\eta =\frac{C_{0}-C_{t}}{C_{0}}\times 100\%$$

where *C*_0_ and *C_t_* are the inlet and outlet concentrations of HCHO, respectively.

In order to quantify the adsorption capacity of BAC on HCHO removal within the dynamic test, Yoon and Nelson (Y-N) model, an approximation method, was applied to nonlinear fitting of breakthrough curves [59]. The Y-N model was expressed as Equation (7):

(7) $$t=\tau +\frac{1}{k^{\prime }}ln\frac{C_{t}}{C_{0}-C_{t}}$$

where *t* is the adsorption time (h), *τ* is the time required for 50% adsorbate breakthrough (h), *k*′ is the rate constant (min^−1^), and *C*_0_ and *C_t_* are the inlet and outlet concentration (mg/m^3^) of the adsorbate, respectively. The catalyst adsorption breakthrough occurred when *C_t_* reached 10% of *C*_0_, and the adsorption saturation occurred when *C_t_* reached 95% of *C*_0_ [60].

Based on the breakthrough curves fitted by Y-N model, the adsorption capacity (*q*) for HCHO was calculated using Equation (8) [61]:

(8) $$q=\frac{F\times C_{0}\times 10^{-6}}{M}\left(T_{s}-\frac{1}{C_{0}}\int_{0}^{\;T_{s}}C_{t}\times dt\right)$$

where *q* is the HCHO amount adsorbed (mg/g), *F* is the flow rate (mL/min), *C*_0_ and *C_t_* are the inlet and outlet concentrations of HCHO, respectively (mg/m^3^), *M* is the mass of adsorbent (g), and *T_s_* is the adsorption saturation time (*C_t_*/*C*_0_ = 0.95, min) ascertained from the breakthrough curves. 

The catalyst with optimal performance for economic applicability was chosen for the regeneration test. Following the dynamic test (Section 2.4.1), the catalyst sample was collected, washed with deionized water 3–5 times, and dried at 100 °C for 12 h. The regenerated catalysts underwent four regeneration experiments, lasting up to 700 min. In addition, humidity was identified as a critical factor affecting catalyst activity during the catalytic process [29]. To simulate the catalyst activity under varying humidity conditions, the gas stream with relative humidity (RH = 0, 25, 75%) was created by adjusting the proportion of dry and humid air.

## 4. Conclusions

In conclusion, a series of BAC@MnO_x_ catalysts were synthesized to degrade indoor HCHO at room temperature. The BAC has natural macropores and develops micropore channels, which are conducive for the homogeneous deposition of catalytic active components through the physical anchoring effect inside/outside the BAC surface. The deposition of catalytic components onto the BAC surface activates reactive oxygen species (O_2_^−^, O^−^). As a result, these oxygen species can participate in the HCHO oxidation reaction at room temperature. Therefore, the as-synthesized BAC@MnO_x_-4 catalyst shows superior HCHO degradation activity, possessing a maximum HCHO removal efficiency of 97% for 17 h without any deactivation (gas hourly space velocity of ~60 L/g_cat_·h) as RH = 0. This study provides a potential approach to develop a promising BAC catalyst for the catalytic oxidative degradation of HCHO at room temperature.

## Figures and Tables

**Table 1:** Pore structural parameters of BAC and series of catalysts.

Sample	*S*_BET_ (m^2^/g)	*S*_micro_ (m^2^/g)	*S*_meso_ (m^2^/g)	*V*_t_ (cm^3^/g)	*V*_micro_ (cm^3^/g)	*V*_micro_/V_t_ (%)	*D*_av_ (nm)
BAC	1061	985	41	0.48	0.39	82.1	—
BAC@MnO_x_-1	879	814	47	0.42	0.33	78.5	1.00
BAC@MnO_x_-2	774	701	39	0.37	0.28	76.3	1.00
BAC@MnO_x_-3	760	702	41	0.35	0.28	79.6	0.92
BAC@MnO_x_-4	783	733	36	0.35	0.29	82.6	0.89
BAC@MnO_x_-5	696	632	45	0.35	0.25	74.0	1.01

*S*_BET_ is specific surface area; *S*_micro_ is micropores surface area; *S*_meso_ is mesopores surface area; *V*_t_ is total pore volume; *V*_micro_ is micropores volume; *D*_av_ is the average pore diameter.

**Table 2:** Surface chemical compositions and related parameters of the series of catalysts.

Sample	Mn 2p_3/2_			O 1s				Mn 3s
	Mn^4+^	Mn^3+^	Mn^3+^/Mn^4+ a^	O’	O_ads_	O_latt_	O_ads_/O_latt_^a^	AOS ^b^
BAC@MnO_x_-1	46.06	53.94	1.17	24.63	48.45	26.92	1.80	3.26
BAC@MnO_x_-2	40.74	59.26	1.45	30.43	43.53	26.04	1.67	3.35
BAC@MnO_x_-3	36.52	63.48	1.74	25.53	46.91	27.55	1.70	3.26
BAC@MnO_x_-4	23.44	76.56	3.27	30.05	46.92	23.03	2.04	3.16
BAC@MnO_x_-5	32.97	67.03	2.03	26.03	47.95	26.01	1.84	3.35

^a^ Mn^3+^/Mn^4+^ and O_ads_/O_latt_ were calculated based on the relative atomic ratio (%) according to Mn 2p and O 1s spectra. ^b^ Average oxidation states (AOS) were calculated by formula: AOS = 8.

**Table 3:** The activity comparison of MnO_x_-based supported catalysts in fixed-bed reactor at room temperature (25 ± 5 °C).

Catalyst	RH (%)	GHSV	Flow Rate (mL/min)	M_c_ (g)	C_0_ (mg/m^3^)	Efficiency (%)	Ref.
BAC@MnO_x_-4	0	60 L/g·h	200	0.2	1.4	η: 97% η_17h_: 97%	This work
	25	60 L/g·h	200	0.2	1.4	η: 98% η_25h_: 97%	
	75	60 L/g·h	200	0.2	1.4	η: 88% η_14h_: 83%	
40%MnO_2_/NCNT	0	30 L/g·h	-	0.2	100 ppm	η: 95%	[21]
3D-MnO_2_	65	90 L/g·h	150	0.1	110 ppm	η: 50%	[8]
GLC-MnO_2_	55	600 L/g·h	1000	0.1	0.5	η: 92% η_10h_: 92%	[52]
MnO_2_/AC	45 ± 5%	120 L/g·h	1000	0.5	0.5	η: 90% η_20h_: 80%	[53]
Ce-MnO_2_	58	600 L/g·h	1000	0.1	500	η: 52% η_6h_: 51%	[54]
MnO_x_/PET	0	17000·h^−1^	1000	0.5	0.6	η: 100% η_10h_: 65%	[55]

## Data Availability

Data are contained within the article and Supplementary Materials.

## Supplementary Materials

The following supporting information can be downloaded at: https://www.mdpi.com/article/10.3390/molecules29030663/s1, Table S1: The Mn contents of BAC@MnO_x_-N (N = 1, 2, 3, 4, 5) series of catalysts; Table S2: The integral areas values of D and G bands of BAC and series of catalysts; Table S3: Binding energy of C 1s, Mn 2p3/2, and O 1s of all catalysts; Table S4: Derived parameters for the Yoon–Nelson fitting of HCHO adsorption on BAC and all catalysts; Figure S1: Schematic diagram of the dynamic test for HCHO adsorption: (a). air compressor; (b). gas dryer; (c). mass flowmeter; (d). bubbler (humid air); (e). water bath; (f). bubbler (HCHO vapor); (g). gas mixing chamber; (h). three-way valve; (i). samples; (j). quartz tube; (k). tail gas treatment; (l). gas gathering system; (m). spectrophotometer instrument; Figure S2: XPS spectra of full survey of BAC and series of catalysts.
