# Supplementary material for: Enhanced and Sustainable Removal of Indoor Formaldehyde by Naturally Porous Bamboo Activated Carbon Supported with MnOx: Synergistic Effect of Adsorption and Oxidation
Source: Molecules. 2024 Jan 31;29(3):663. doi: 10.3390/molecules29030663 (PMC10856013; doi:10.3390/molecules29030663)
Supplement: Supplementary file 1 [file molecules-29-00663-s001.zip › molecules-2831995-supplementary.pdf]

Supplementary Material for

Enhanced and Sustainable Removal of Indoor Formaldehyde by Naturally Porous Bamboo Activated Carbon Supported with MnO_x: Synergistic Effect of Adsorption and Oxidation

Zhenrui Li [1,2], Yujun Li [1,2], Shijie Li [1,2], Jianfeng Ma [1,2], Qianli Ma [1,2], Zhihui Wang [1,2], Jiajun Wang [1,2], Keying Long [3] and Xing'e Liu [1,2,*]

[1] International Centre for Bamboo and Rattan, Beijing 100102, China; lizr@caf.ac.cn (Z.L.); liyujun0225@163.com (Y.L.); lishijie@vision-smart.com (S.L.); majf@icbr.ac.cn (J.M.); maql@icbr.ac.cn (Q.M.); frankie2499@163.com (Z.W.); wjjchina0112@163.com (J.W.)
[2] Key Laboratory of National Forestry and Grassland Administration/Beijing for Bamboo & Rattan Science and Technology, Beijing 100102, China
[3] Guangxi Zhuang Autonomous Region Forestry Research Institute, Nanning 530002, China; longky0305@163.com
* Correspondence: liuxinge@icbr.ac.cn; Tel.: +86-139-1067-7147

The supplementary material contains 5 pages, including Tables S1-S4, and Figures S1-S2.

1. Supporting Tables

Table S1. The Mn contents of BAC@MnO$_x$-N (N = 1, 2, 3, 4, 5) series of catalysts.

Samples	BAC@MnO$_x$-1	BAC@MnO$_x$-2	BAC@MnO$_x$-3	BAC@MnO$_x$-4	BAC@MnO$_x$-5
Mn (wt.%)	7.72	8.98	10.24	10.44	11.04

Table S2. The integral areas values of D and G bands of BAC and series of catalysts.

Samples	I_D	I_G	I_D/I_G
BAC	417829.2	171227.4	2.44
BAC@MnO$_x$-1	147776.3	63541.4	2.33
BAC@MnO$_x$-2	354249.7	148978.5	2.38
BAC@MnO$_x$-3	71912.9	31094.2	2.31
BAC@MnO$_x$-4	51459.5	21352.5	2.41
BAC@MnO$_x$-5	107913.9	45252.3	2.38

Table S3. Binding energy of C 1s, Mn 2p3/2, and O 1s of all catalysts.

Samples	Binding energy (eV)							
	C 1s			Mn 2p3/2		O 1s		
	sp^2 C=C	sp^3 C-C	C=O	Mn^{3+}	Mn^{4+}	O’	O_{ads}	O_{latt}
BAC@MnO$_x$-1	284.8	285.3	288.1	642.1	643.6	533.5	531.9	530.1
BAC@MnO$_x$-2	284.8	285.6	289.0	642.1	643.8	533.6	532.1	530.0
BAC@MnO$_x$-3	284.8	285.3	288.3	642.2	643.9	533.4	531.7	530.0
BAC@MnO$_x$-4	284.8	285.3	288.3	642.3	644.6	533.4	531.7	530.0
BAC@MnO$_x$-5	284.8	285.3	288.3	642.3	644.2	533.4	531.6	530.0

Table S4. Derived parameters for the Yoon-Nelson fitting of HCHO adsorption on BAC and all catalysts.

Samples	C_0 (mg·m^{-3})	K_{YN} (min^{-1})	τ (h)	τ_{exp} (h)	R^2
**BAC**	1.439	1.518	2.608	2.703	0.993
**BAC@MnO$_x$-1**	1.423	0.326	27.814	29.041	0.992
**BAC@MnO$_x$-2**	1.415	0.116	47.298	51.802	0.995
**BAC@MnO$_x$-3**	1.398	0.092	56.659	61.392	0.991
**BAC@MnO$_x$-4**	1.407	0.088	62.558	67.568	0.990
**BAC@MnO$_x$-5**	1.446	0.083	63.137	66.210	0.990

2. Supporting Figures

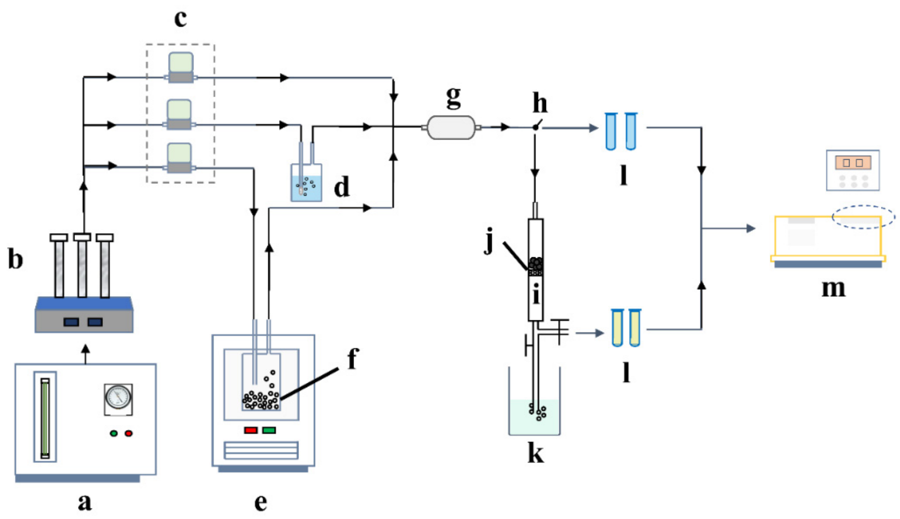

Figure S1. Schematic diagram of the dynamic test for HCHO adsorption: a. air compressor; b. gas dryer; c. mass flowmeter; d. bubbler (humid air); e. water bath; f. bubbler (HCHO vapor); g. gas mixing chamber; h. three-way valve; i. samples; j. quartz tube; k. tail gas treatment; l. gas gathering system; m. spectrophotometer instrument.

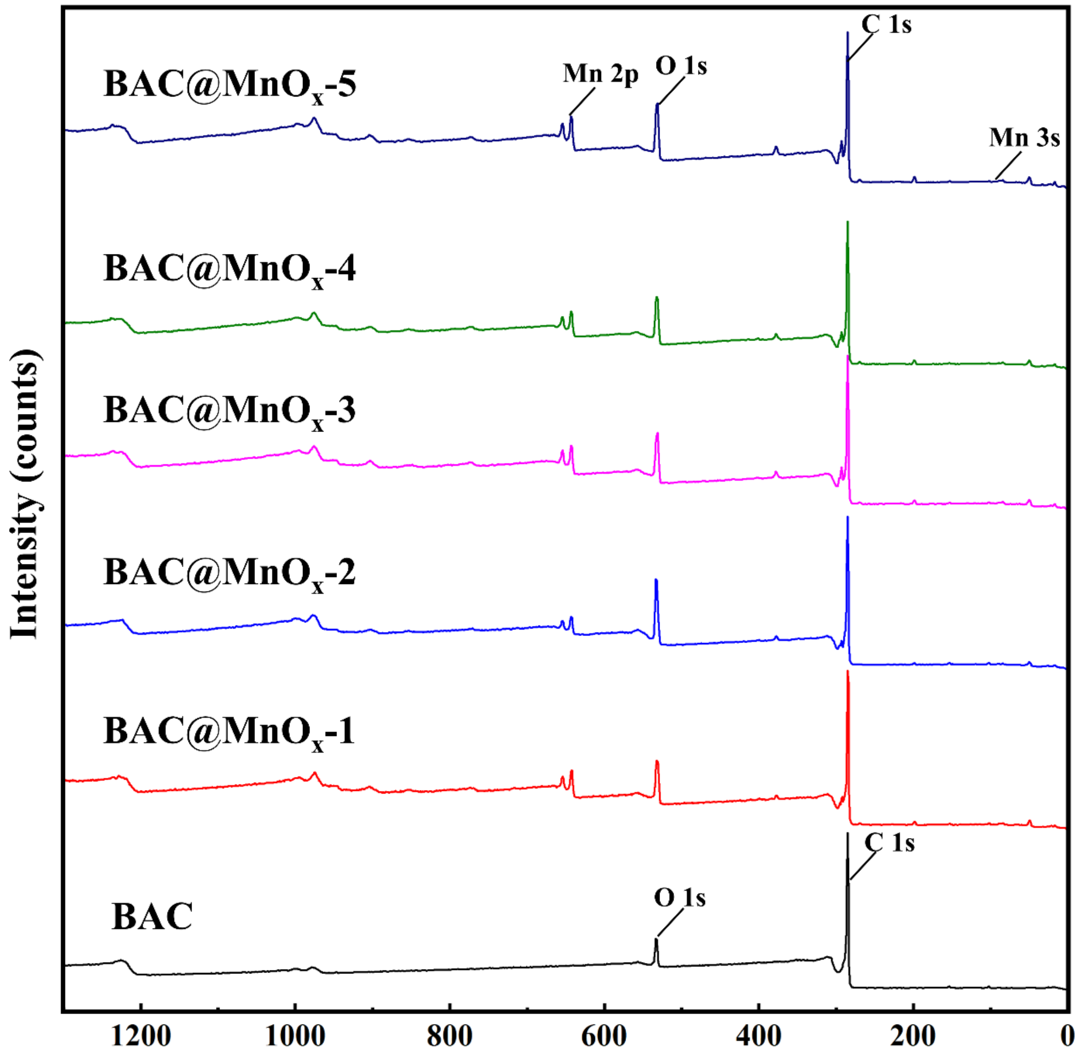

Figure S2. XPS spectra of full survey of BAC and series of catalysts.
